# Supplementary material for: Biomarker discovery in inflammatory bowel diseases using network-based feature selection
Source: PLoS One. 2019 Nov 22;14(11):e0225382. doi: 10.1371/journal.pone.0225382 (PMC6874333; doi:10.1371/journal.pone.0225382)
Supplement: S2 File — (DOCX) [file pone.0225382.s002.docx]

| 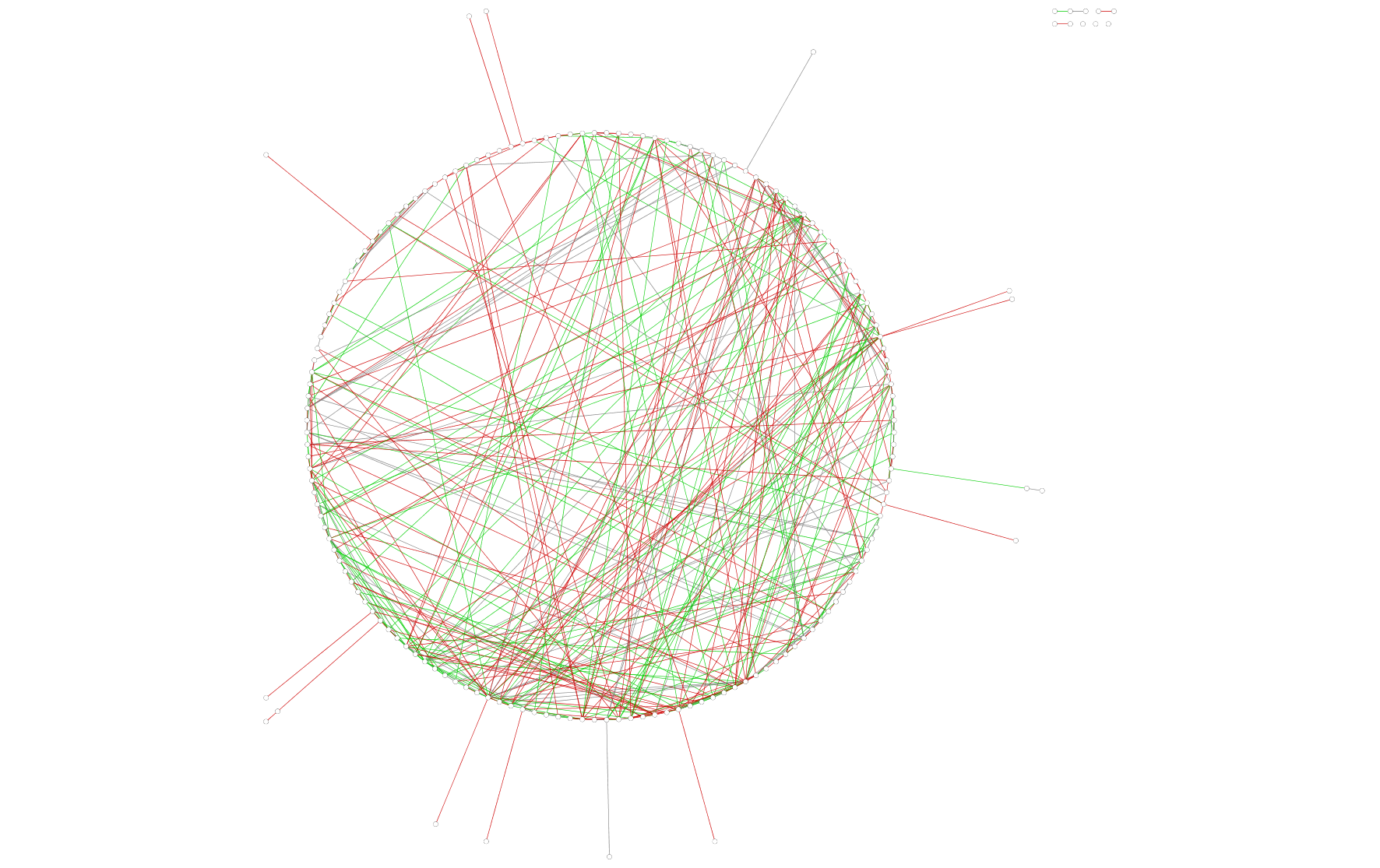 |
| --- |
| 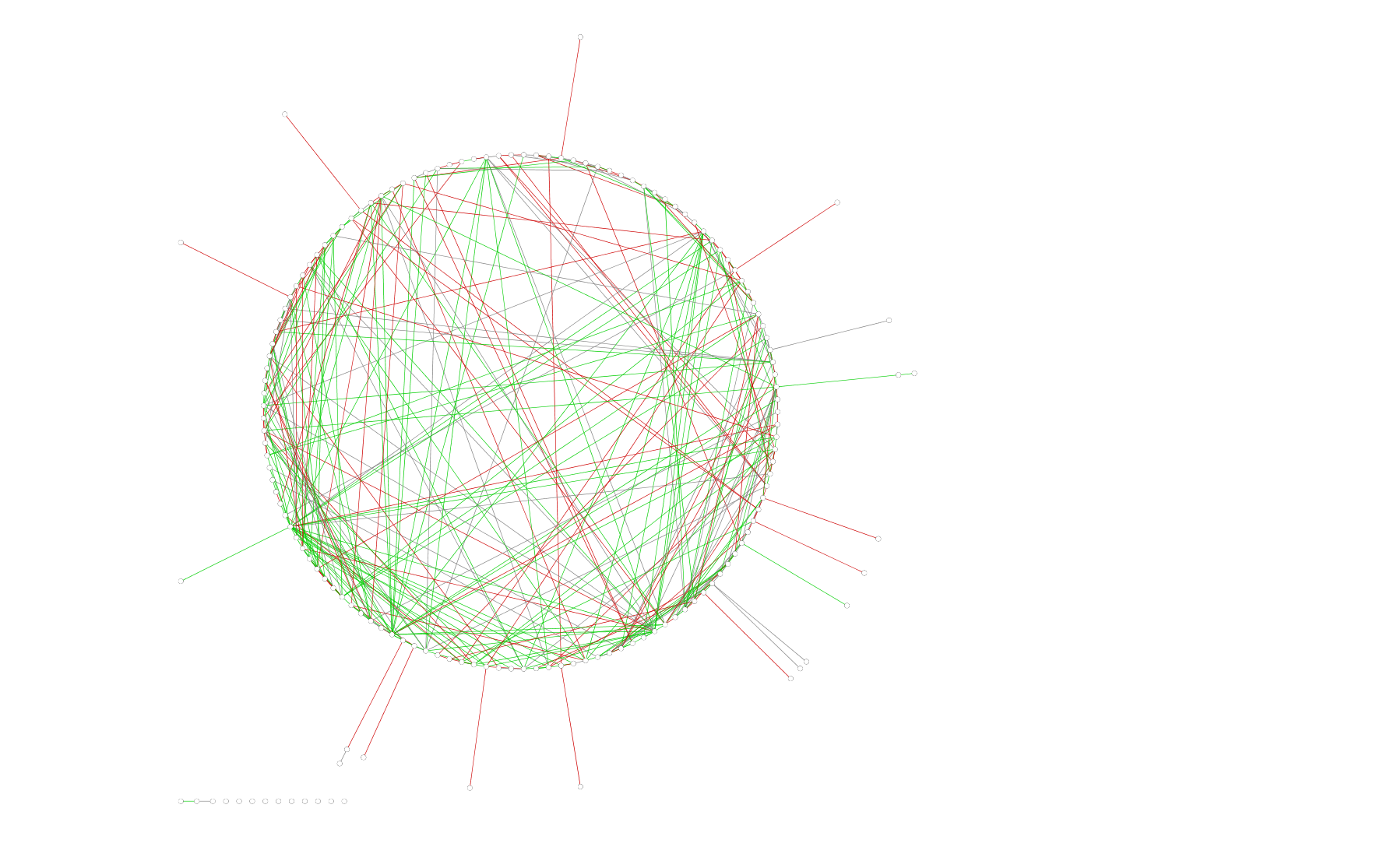 |

**Supplementary Figure A: Comparisons between IBD (left) and healthy (right) networks inferred using MB tool applied to FSDS50 (green) and FSDS400 (red). Gray edges represent common edges between the two compared networks.**


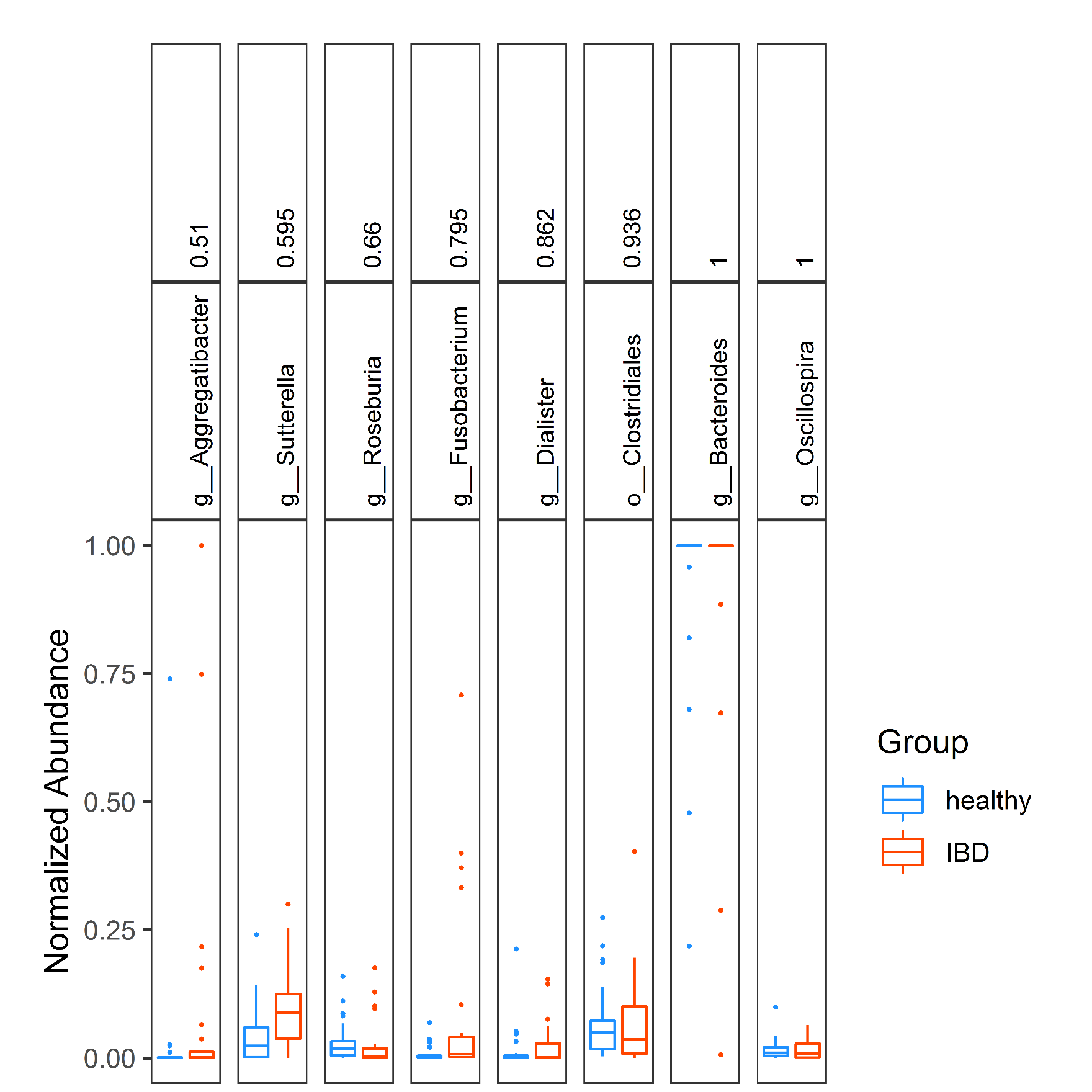


**Supplementary Figure B: Box plots of normalized abundance for the eight common IBD biomarkers including p-values obtained using Kruskal-Wallis test of medians applied to FSDS50**


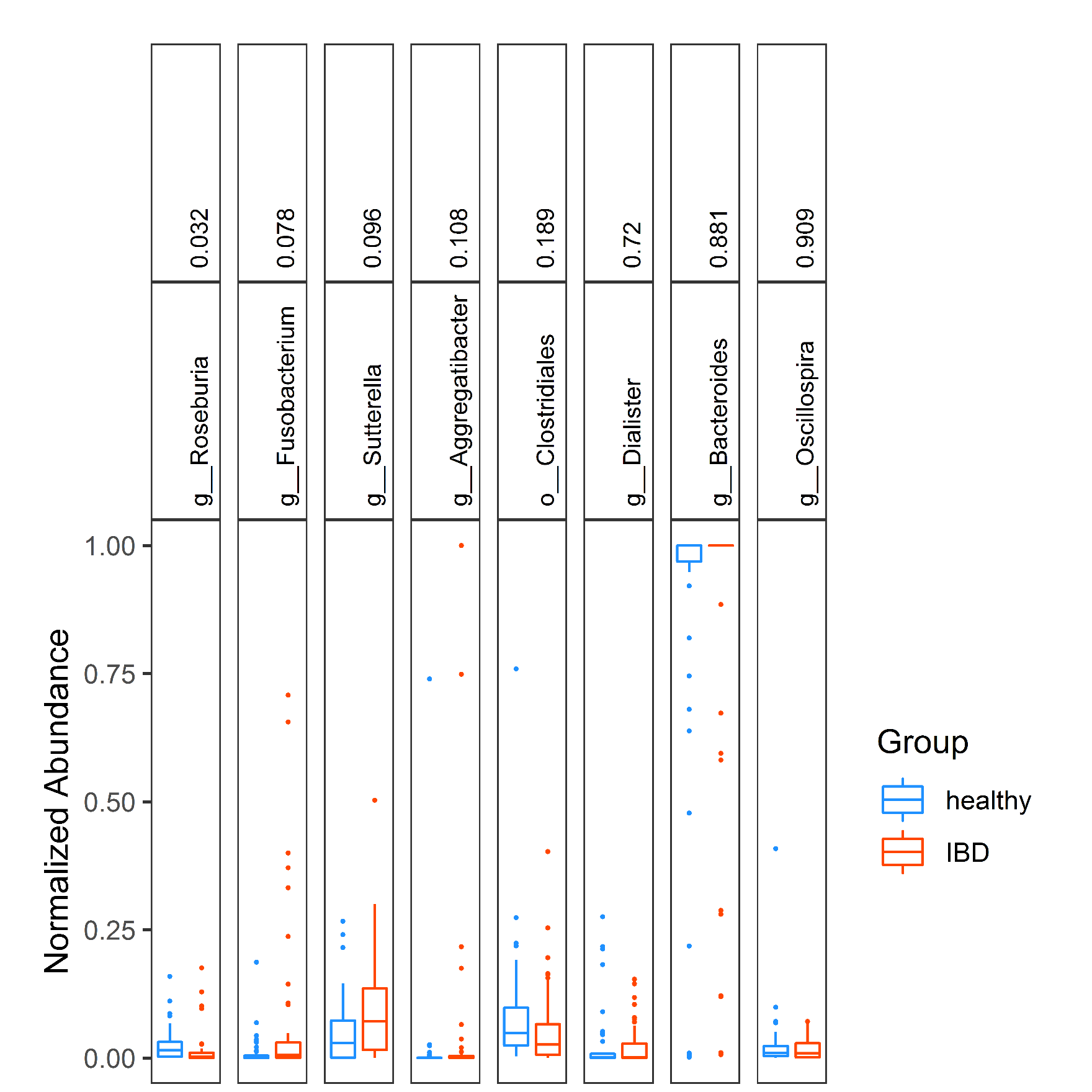


**Supplementary Figure C: Box plots of normalized abundance for the eight common IBD biomarkers including p-values obtained using Kruskal-Wallis test of medians applied to FSDS100**


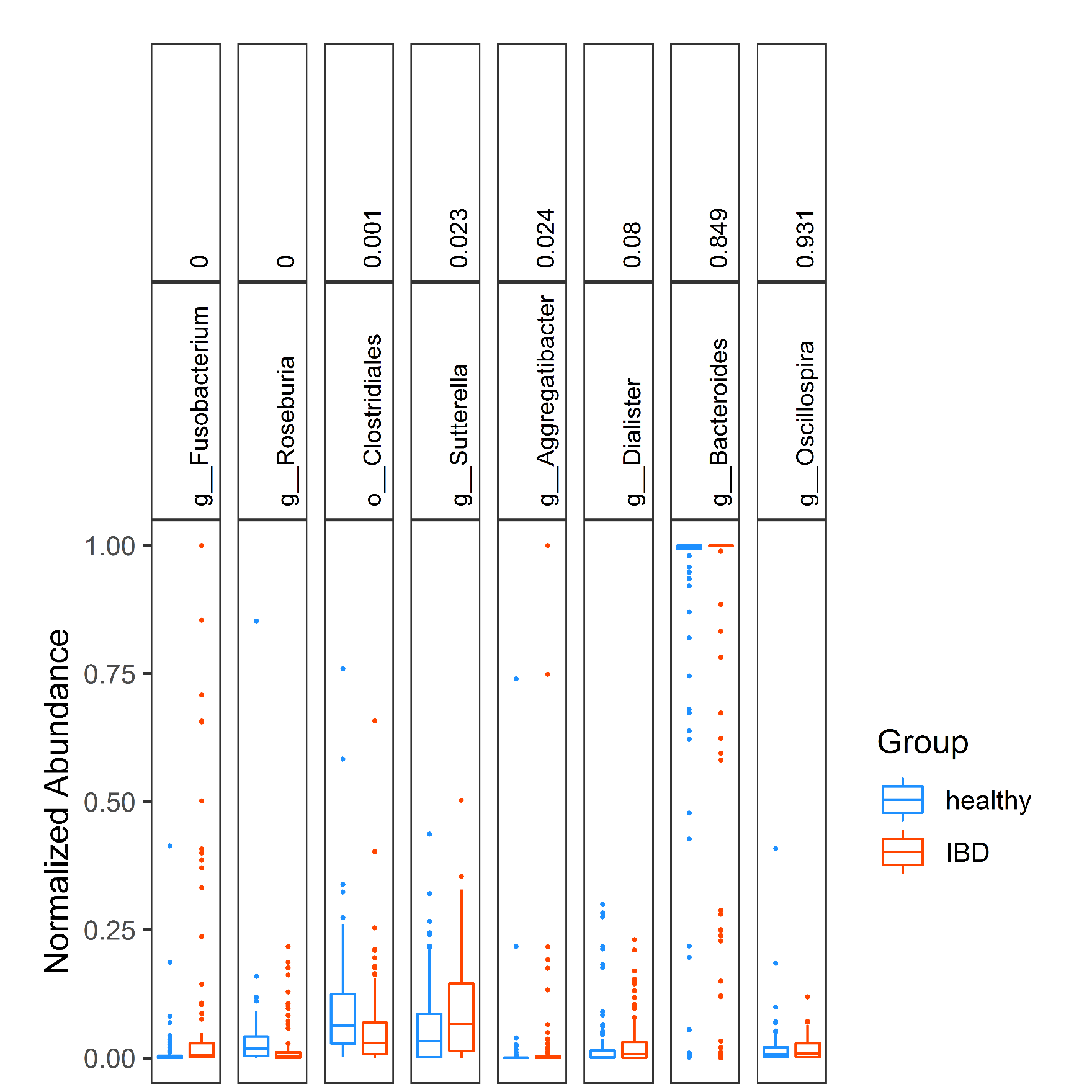


**Supplementary Figure D: Box plots of normalized abundance for the eight common IBD biomarkers including p-values obtained using Kruskal-Wallis test of medians applied to FSDS200**


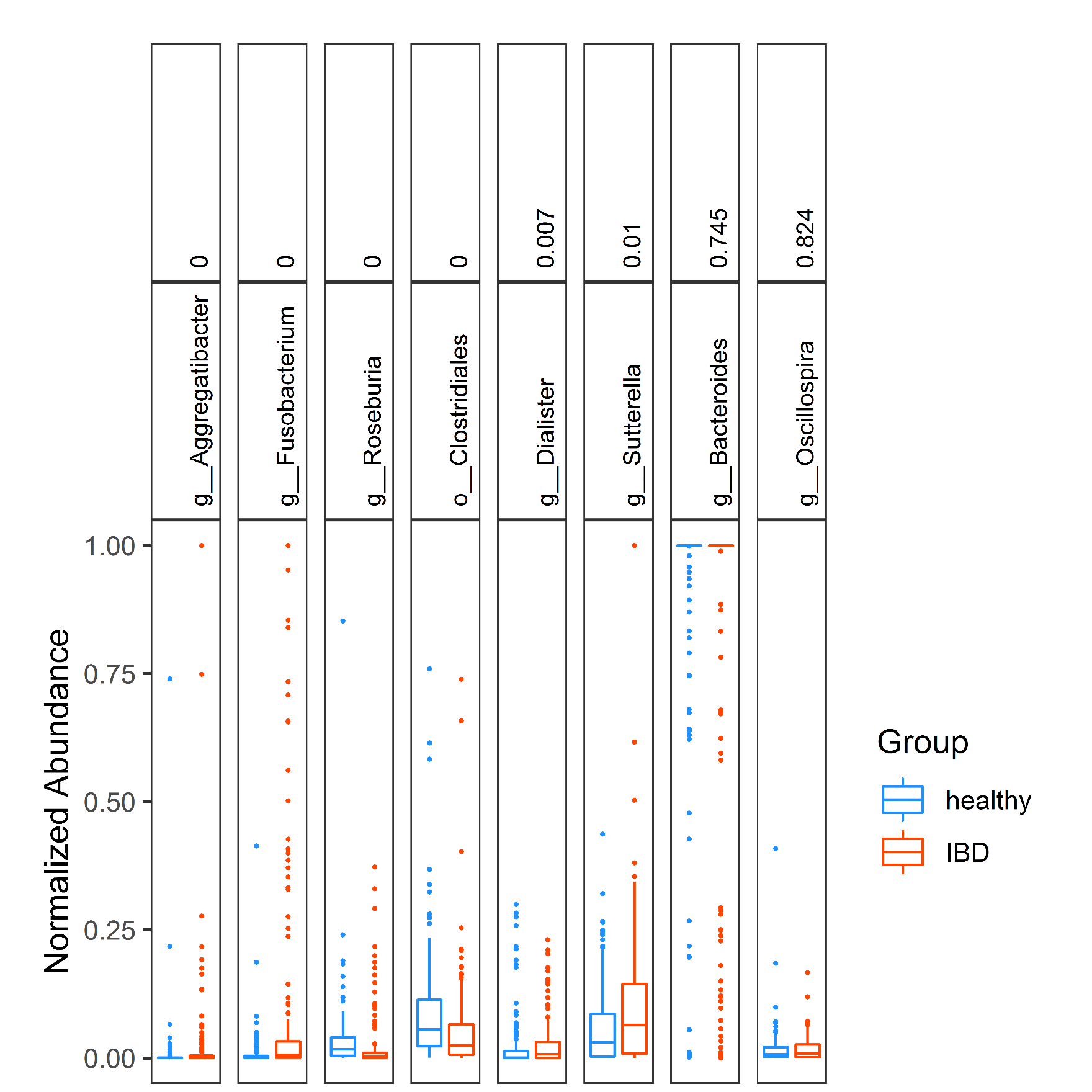


**Supplementary Figure E: Box plots of normalized abundance for the eight common IBD biomarkers including p-values obtained using Kruskal-Wallis test of medians applied to FSDS300**


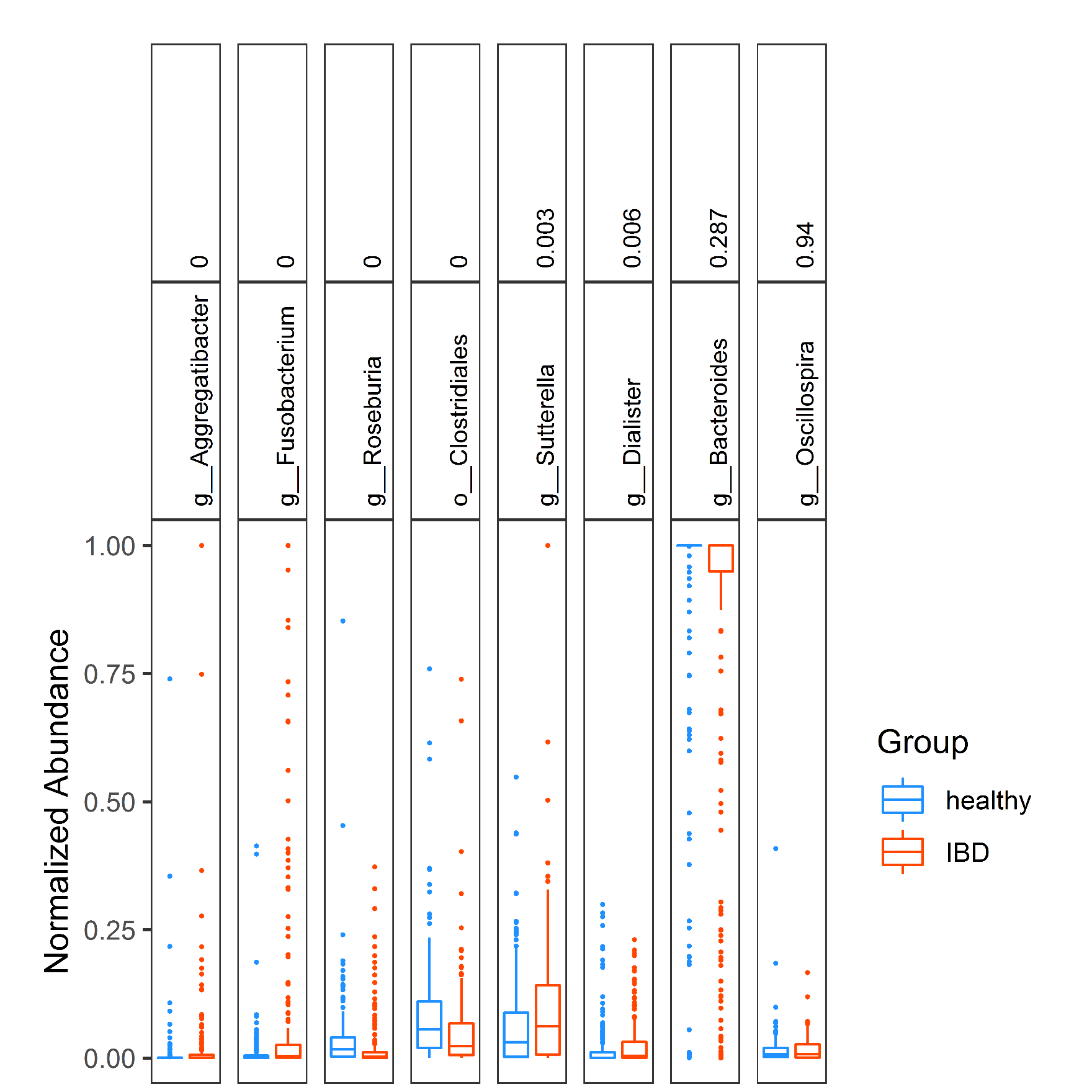


**Supplementary Figure F: Box plots of normalized abundance for the eight common IBD biomarkers including p-values obtained using Kruskal-Wallis test of medians applied to FSDS400**


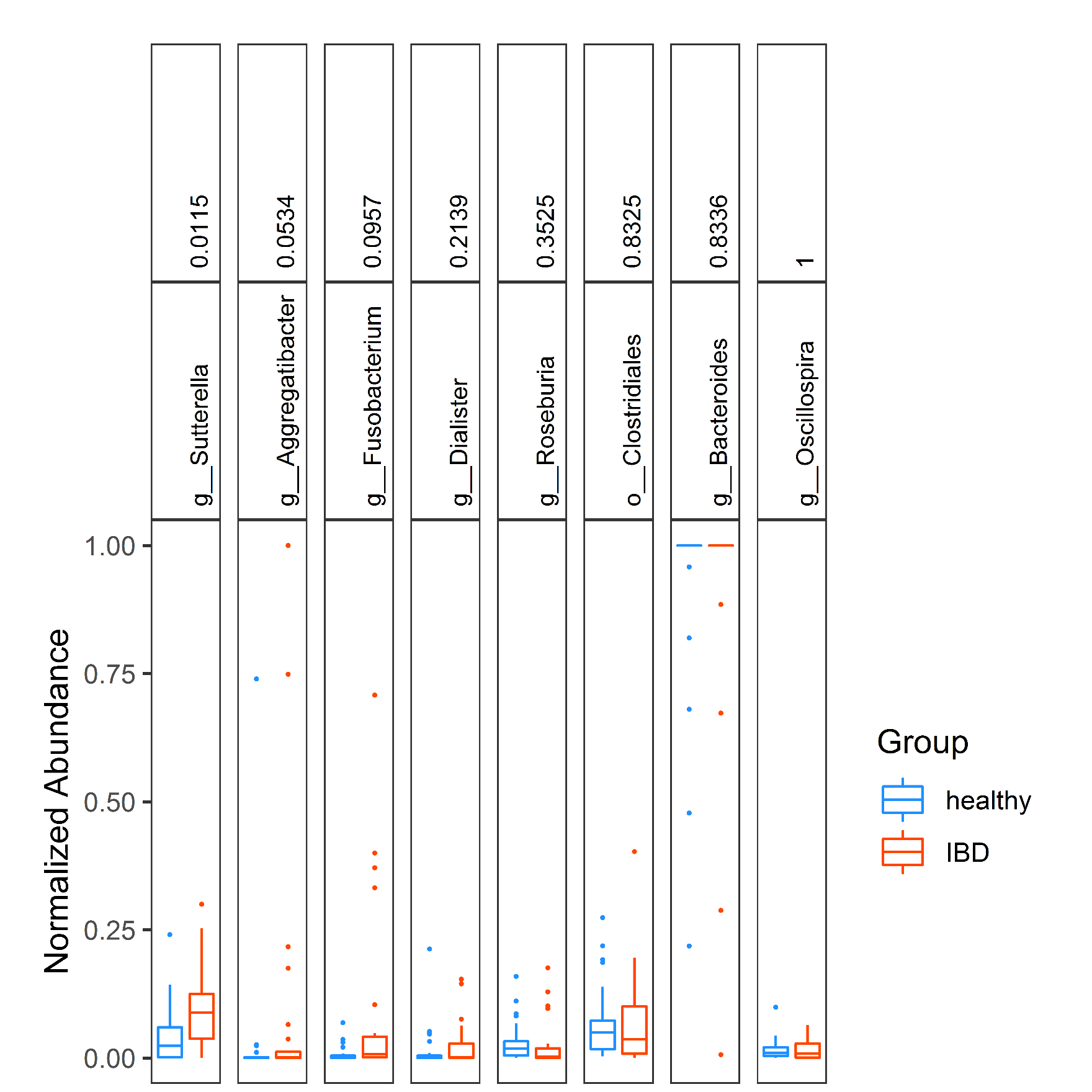


**Supplementary Figure G: Box plots of normalized abundance for the eight common IBD biomarkers including p-values obtained using Mann-Whitney test of medians applied to FSDS50**


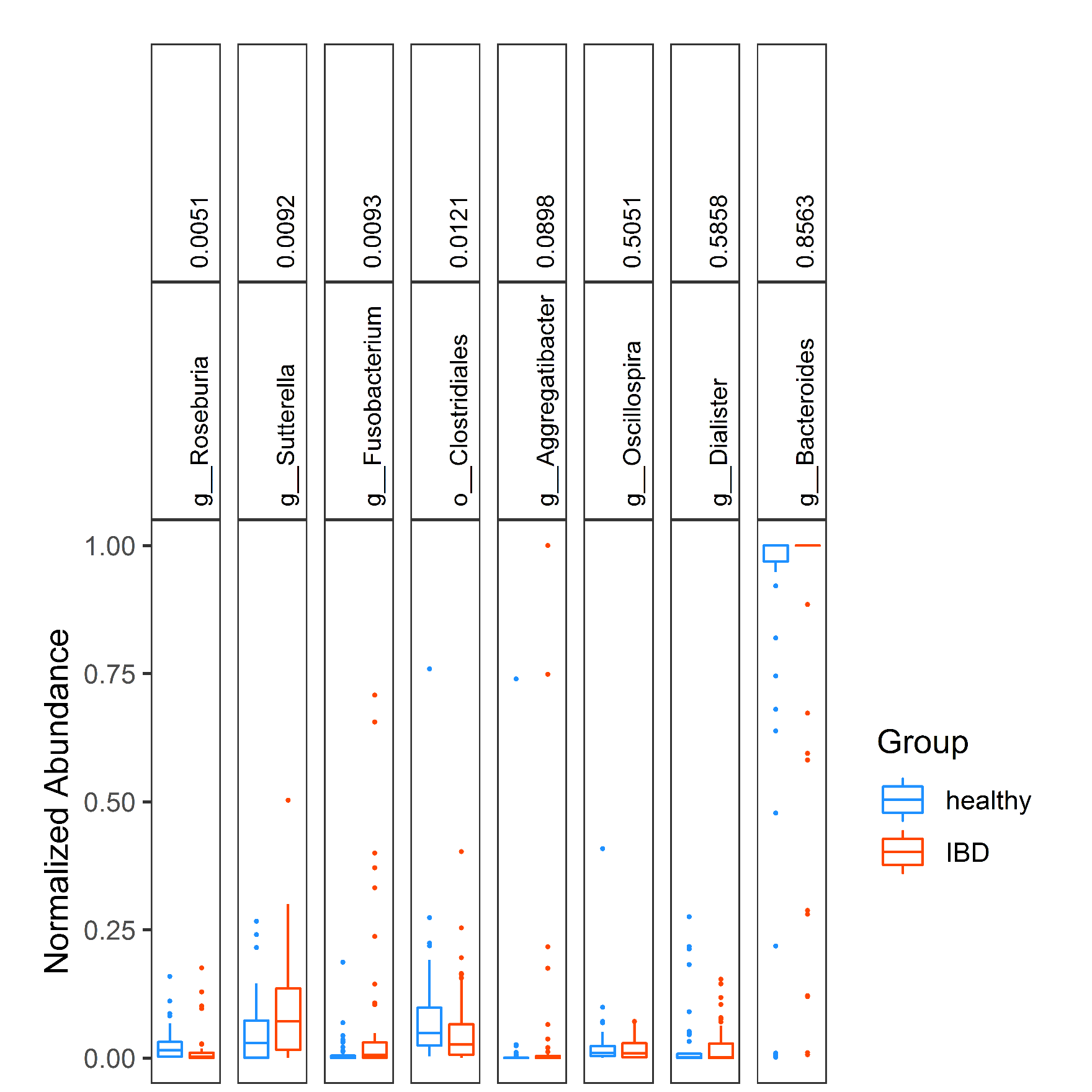


**Supplementary Figure H: Box plots of normalized abundance for the eight common IBD biomarkers including p-values obtained using Mann-Whitney test of medians applied to FSDS100**


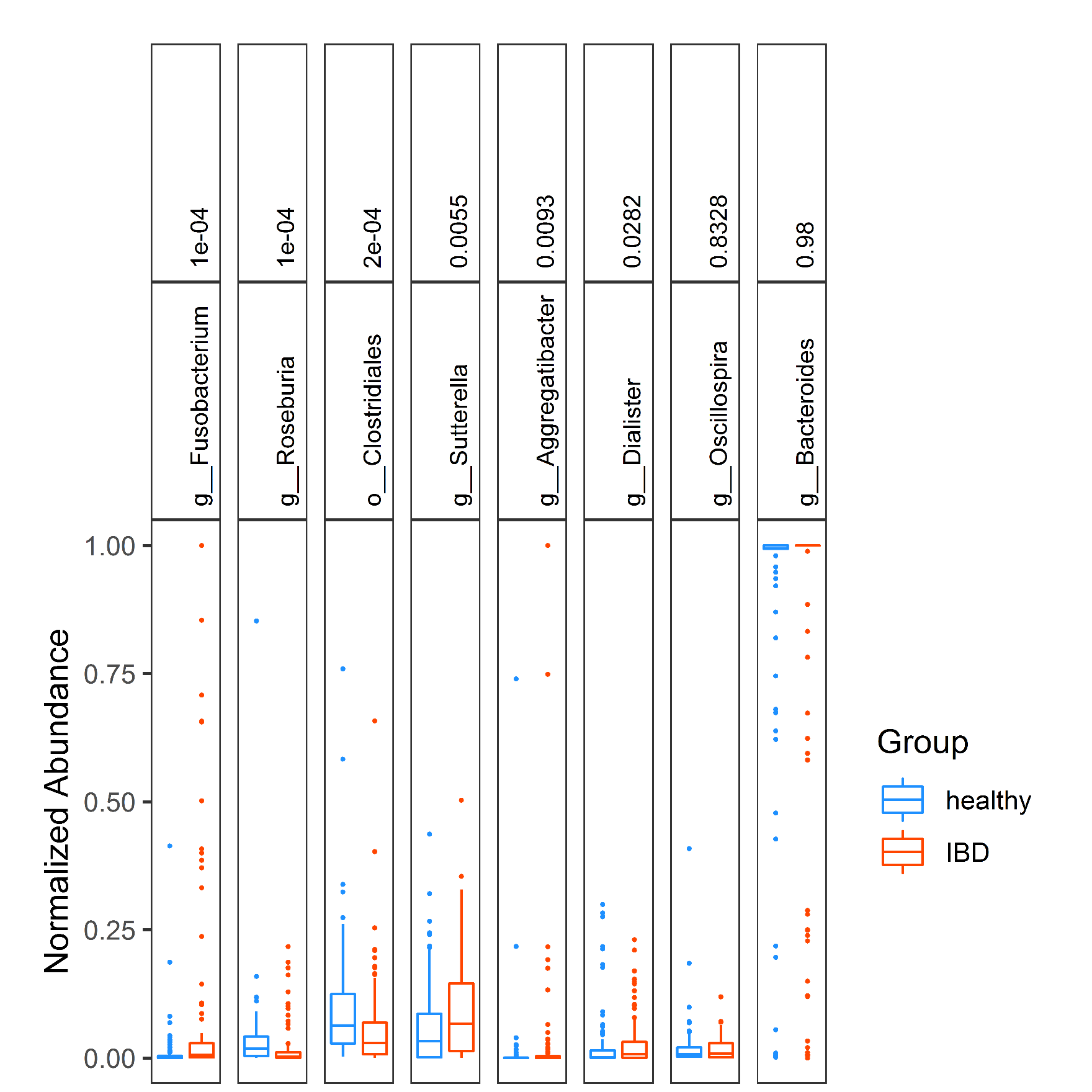


**Supplementary Figure I: Box plots of normalized abundance for the eight common IBD biomarkers including p-values obtained using Mann-Whitney test of medians applied to FSDS200**


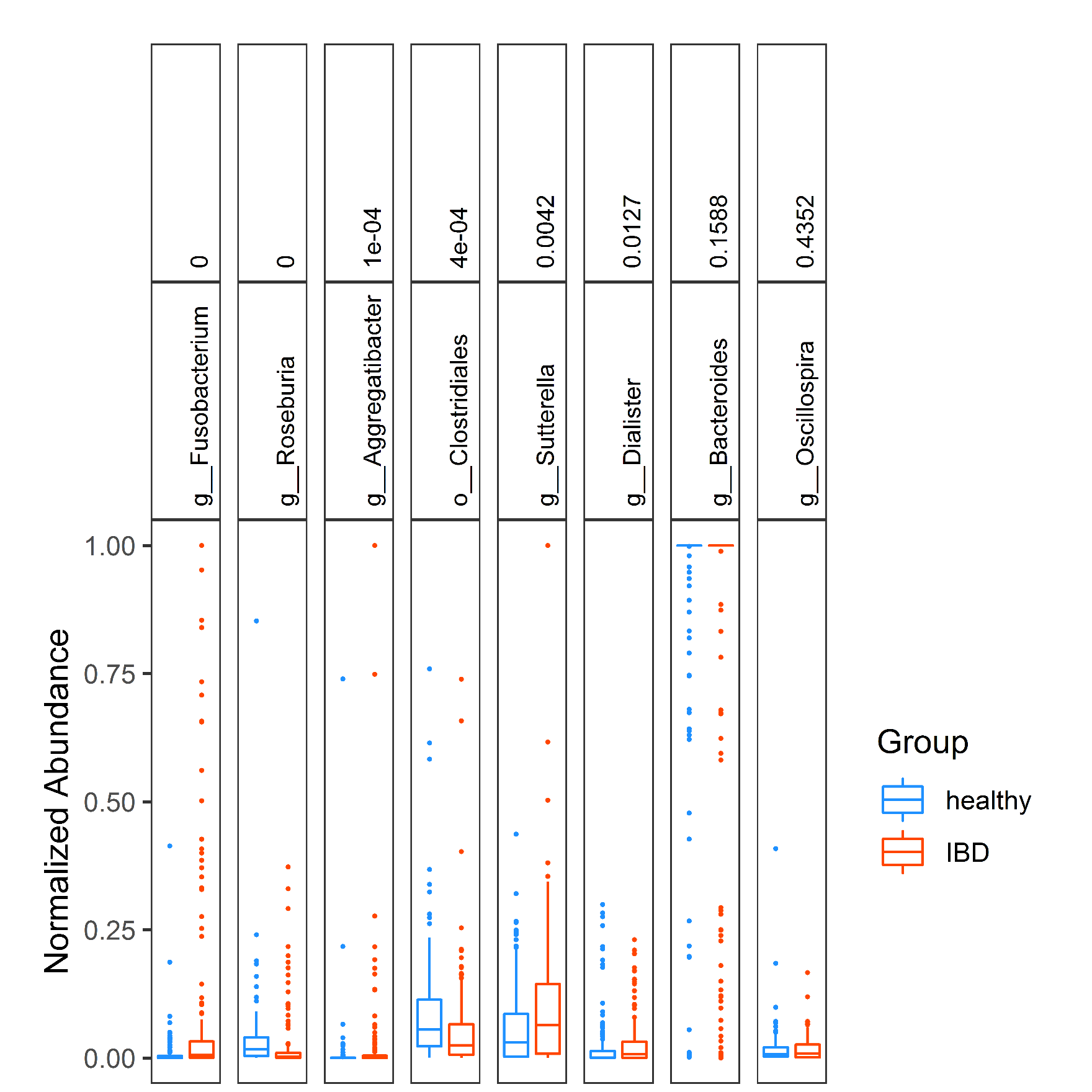


**Supplementary Figure J: Box plots of normalized abundance for the eight common IBD biomarkers including p-values obtained using Mann-Whitney test of medians applied to FSDS300**


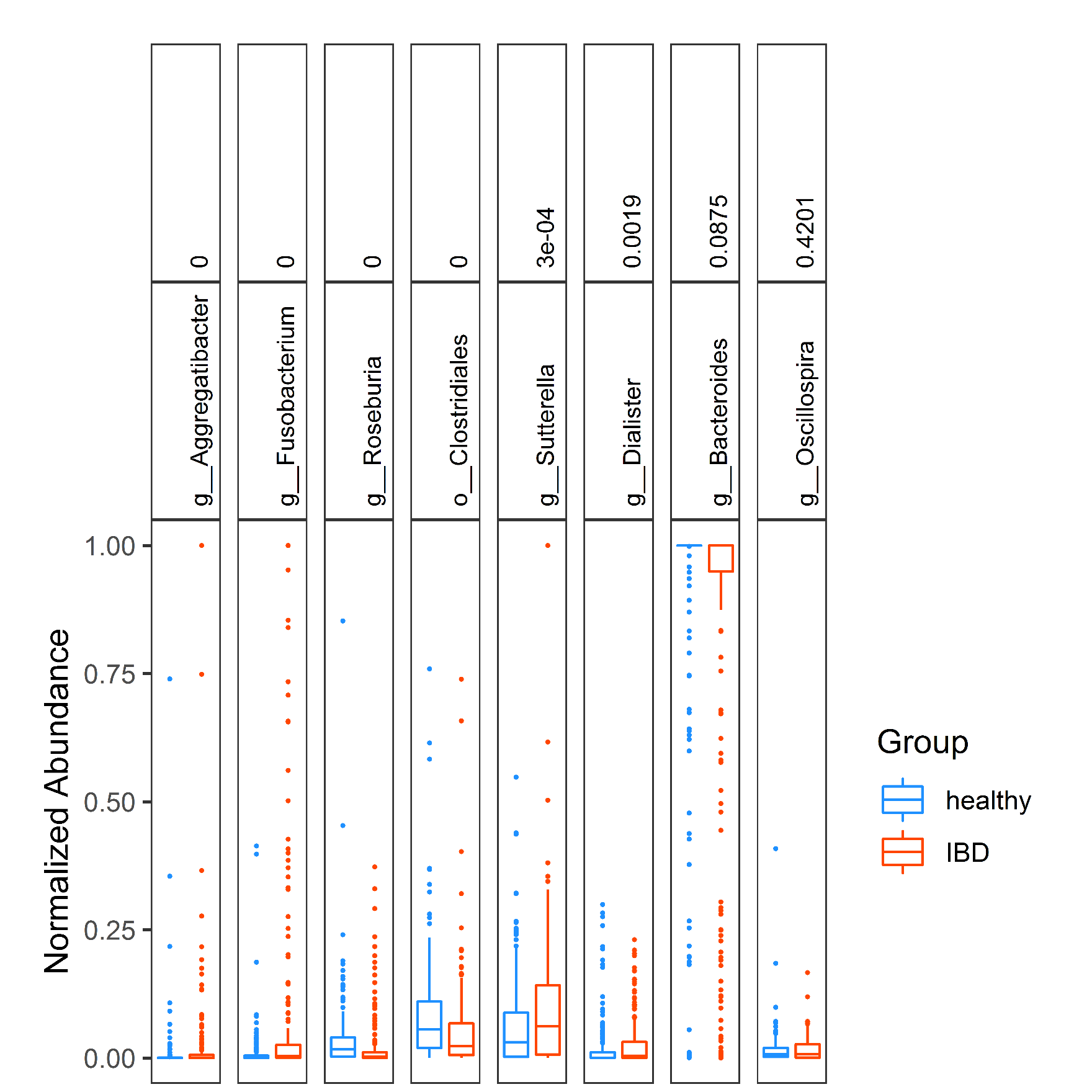


**Supplementary Figure K: Box plots of normalized abundance for the eight common IBD biomarkers including p-values obtained using Mann-Whitney test of medians applied to FSDS400**


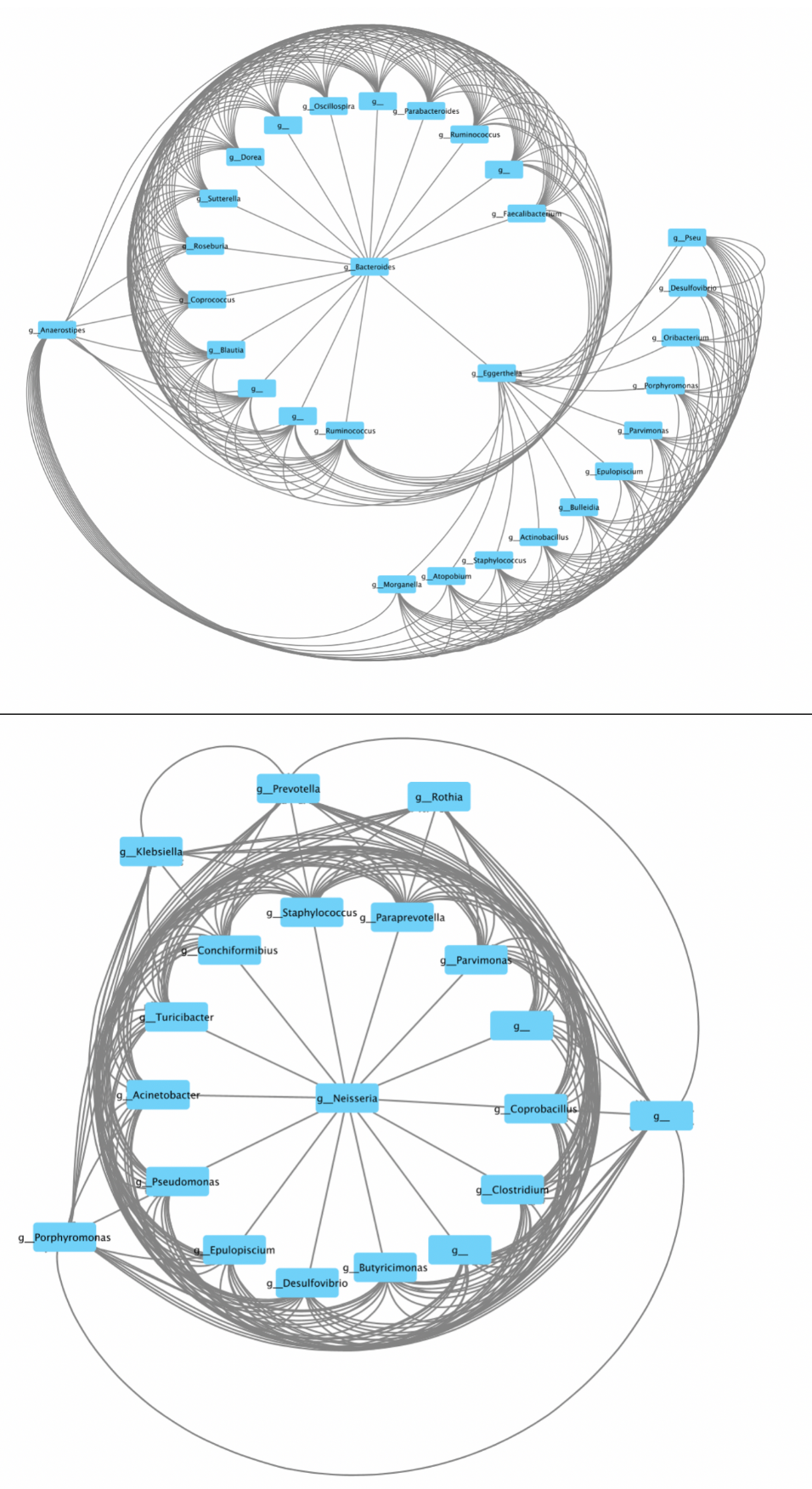


**Supplementary Figure L: Top highly connected module in IBD (top) and healthy (bottom) networks inferred using the RMT tool and the DS400 dataset.**


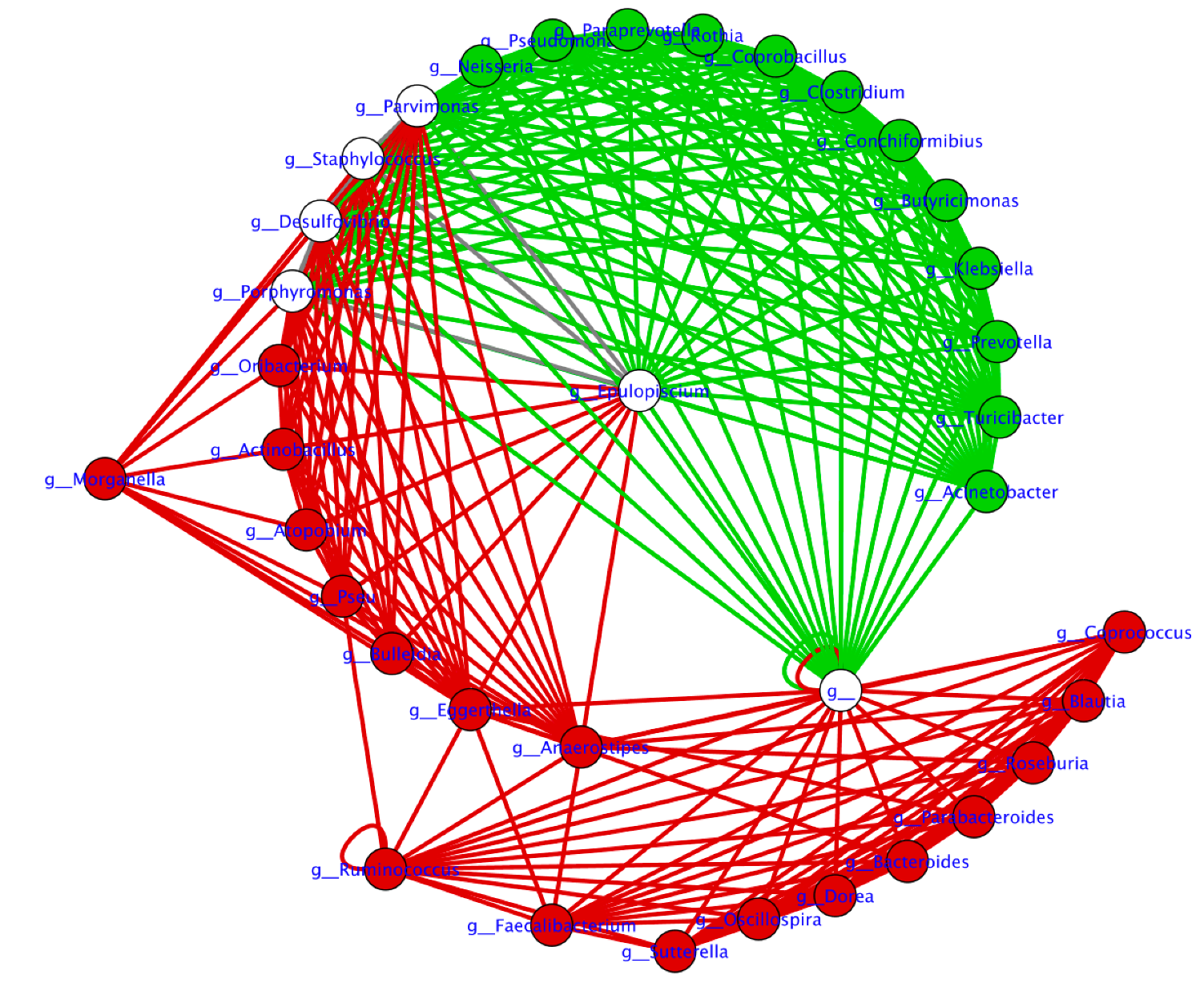


**Supplementary Figure M: DyNet visualization of the differences between the top IBD (red) and healthy (green) modules. Shared nodes and edges are highlighted in white and gray, respectively.**


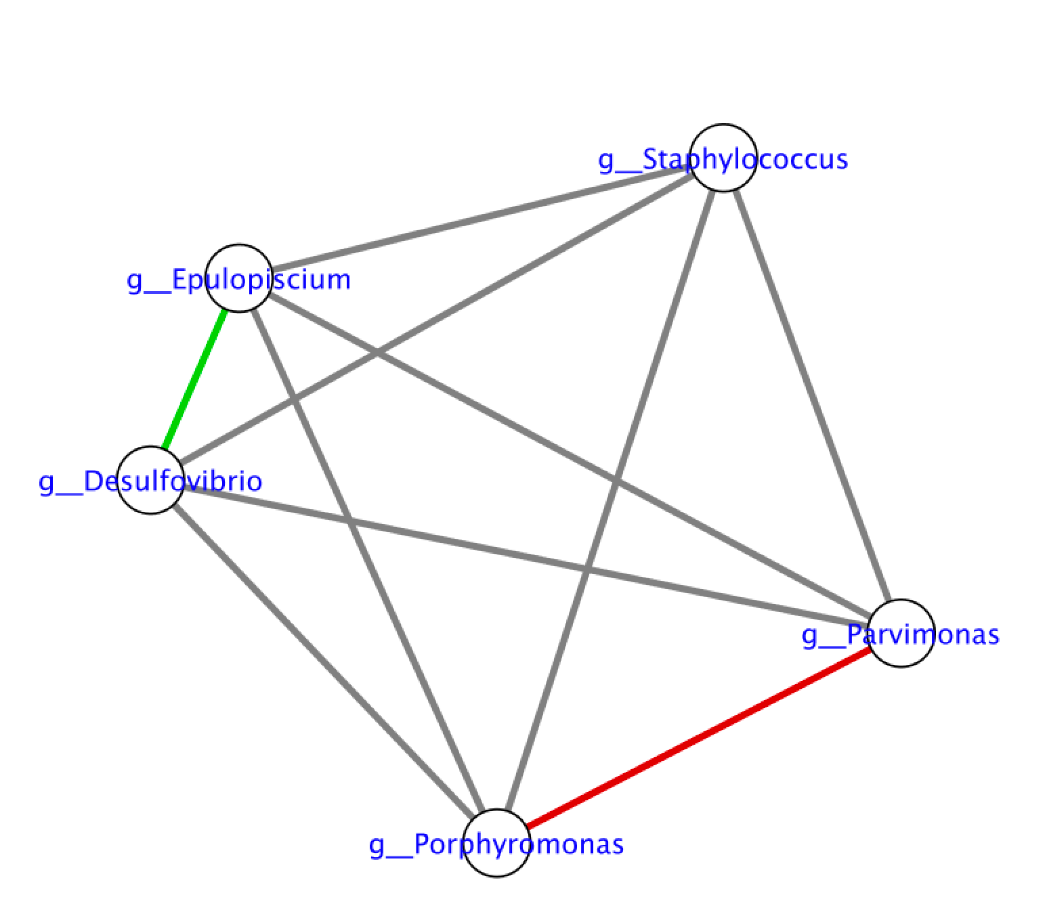


**Supplementary Figure N: Sub-network comprised of common nodes in Fig. S13 and their interactions.**


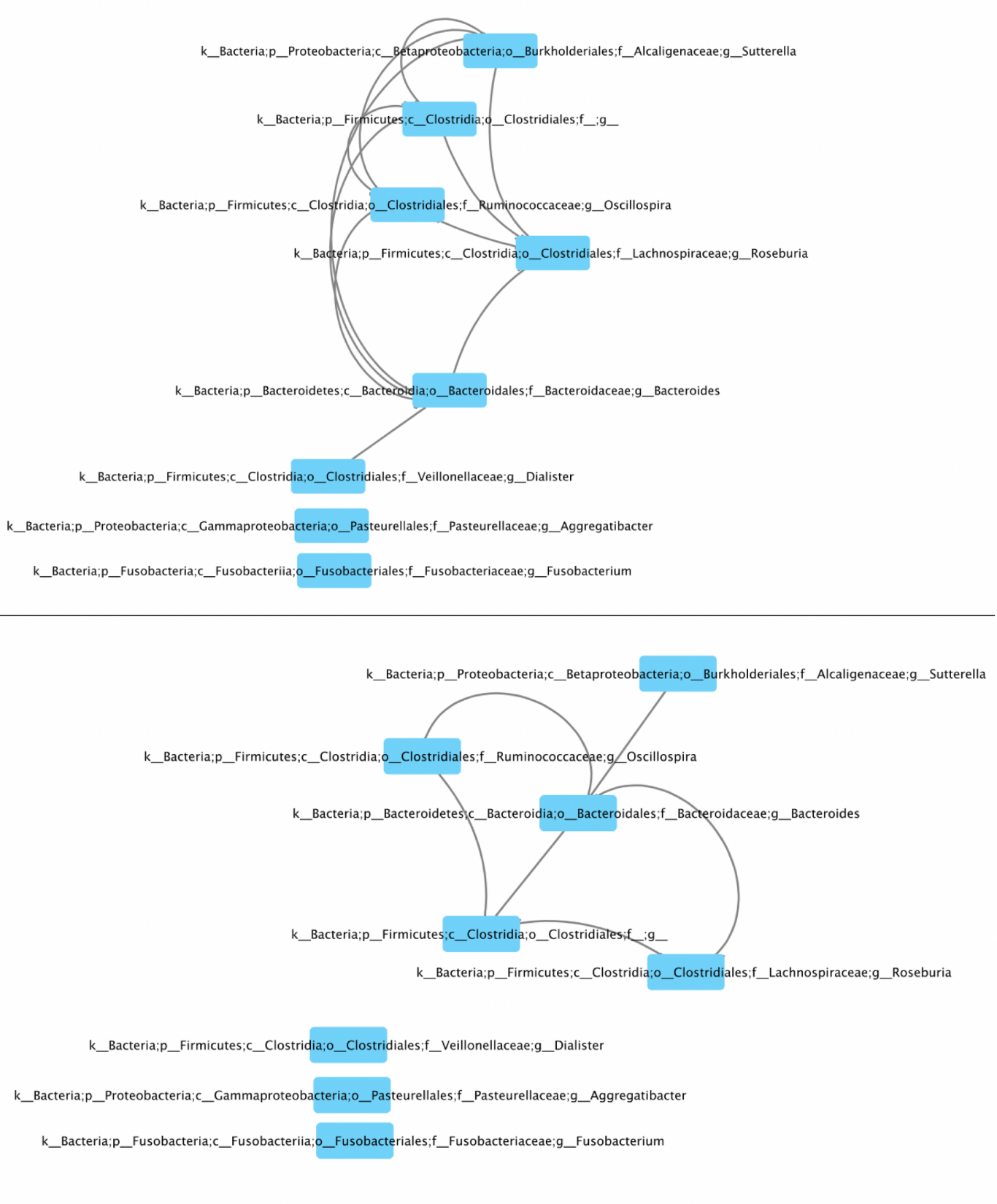


**Supplementary Figure O: Biomarkers sub-networks extracted from IBD (top) and healthy (bottom) networks and inferred using the RMT tool and the DS320 dataset.**
